# *Pseudomonas aeruginosa* esterase PA2949, a bacterial homolog of the human membrane esterase ABHD6: expression, purification and crystallization

**DOI:** 10.1107/S2053230X19002152

**Published:** 2019-04-02

**Authors:** Florian Bleffert, Joachim Granzin, Holger Gohlke, Renu Batra-Safferling, Karl-Erich Jaeger, Filip Kovacic

**Affiliations:** a Institute of Molecular Enzyme Technology, Heinrich-Heine-Universität Düsseldorf, Forschungszentrum Jülich GmbH, D-52426 Jülich, Germany; b Institute of Complex Systems ICS-6: Structural Biochemistry, Forschungszentrum Jülich GmbH, D-52425 Jülich, Germany; c Institute of Pharmaceutical and Medicinal Chemistry, Heinrich-Heine-Universität Düsseldorf, D-40225 Düsseldorf, Germany; d John von Neumann Institute for Computing (NIC) and Jülich Supercomputing Centre (JSC), Forschungszentrum Jülich GmbH, D-52425 Jülich, Germany; e Institute of Bio- and Geosciences IBG-1: Biotechnology, Forschungszentrum Jülich GmbH, D-52426 Jülich, Germany

**Keywords:** *Pseudomonas aeruginosa*, PA2949, esterases, ABHD6, endocannabinoid signalling pathway, Parkinson’s disease, Alzheimer’s disease

## Abstract

The human membrane-bound α/β-hydrolase domain 6 (ABHD6) protein modulates endocannabinoid signaling, which controls appetite, pain and learning, as well as being linked to Alzheimer’s and Parkinson’s diseases, through the degradation of the key lipid messenger 2-arachidonylglycerol (2-AG). This makes ABHD6 an attractive therapeutic target that lacks structural information. In order to better understand the molecular mechanism of 2-AG-hydrolyzing enzymes, the PA2949 protein from *Pseudomonas aeruginosa*, which has 49% sequence similarity to the ABHD6 protein, was cloned, overexpressed, purified and crystallized. Overexpression of PA2949 in the homologous host yielded the membrane-bound enzyme, which was purified in milligram amounts. Besides their sequence similarity, the enzymes both show specificity for the hydrolysis of 2-AG and esters of medium-length fatty acids. PA2949 in the presence of *n*-octyl β-d-glucoside showed a higher activity and stability at room temperature than those previously reported for PA2949 overexpressed and purified from *Escherichia coli*. A suitable expression host and stabilizing detergent were crucial for obtaining crystals, which belonged to the tetragonal space group *I*4_1_22 and diffracted to a resolution of 2.54 Å. This study provides hints on the functional similarity of ABHD6-like proteins in prokaryotes and eukaryotes, and might guide the structural study of these difficult-to-crystallize proteins.

## Abstract 2

Homologous expression of the membrane-bound esterase PA2949 from *Pseudomonas aeruginosa* PA01 and the purification of detergent-solubilized enzyme resulted in stable PA2949 protein that crystallized. The crystals obtained were used for X-ray analysis and diffracted to a resolution of 2.74 Å.

## 1. Introduction   

Enzymes of the α/β-hydrolase superfamily are found in virtually all organisms and have functional implications that are important to human health (Lord *et al.*, 2013 ▸) and bacterial pathogenesis (Flores-Díaz *et al.*, 2016 ▸). The canonical α/β-hydrolase fold consists of up to 11 β-strands folded into a central hydrophobic sheet surrounded by flanking α-helices (Heikinheimo *et al.*, 1999 ▸). This fold provides a scaffold for a structurally conserved active site comprising the catalytic triad (Ser, His, Asp) and two oxyanion-hole residues (Heikinheimo *et al.*, 1999 ▸). The hydrolytic reactions that are catalyzed by α/β-hydrolases rely on a hydrogen-bond network between the catalytic triad residues that is essential for formation of the nucleophilic serine (Rauwerdink & Kazlauskas, 2015 ▸). The residues of the oxyanion hole stabilize the tetrahedral intermediates (Pérez *et al.*, 2012 ▸; Rauwerdink & Kazlauskas, 2015 ▸).

Mammals express at least 19 α/β-hydrolases [called α/β-hydrolase domain (ABHD) proteins in the literature], and the biochemical and physiological functions of most of them are largely unknown (Lord *et al.*, 2013 ▸). Functional proteomics of brain tissue revealed ABHD6 to be a 2-arachidonylglycerol (2-AG) hydrolase (Blankman *et al.*, 2007 ▸). 2-AG is biosynthesized from membrane phospholipid precursors and stimulates cannabinoid (CB) receptors in the vicinity of its own synthesis. A recent study confirmed that ABHD6 controls the levels and signaling efficacy of 2-AG in neurons and is thus considered to be a member of the endocannabinoid signaling pathway (Marrs *et al.*, 2010 ▸). This system controls diverse physiological processes such as pain sensation, the maintenance of food intake, learning and memory, and is related to Alzheimer’s and Parkinson’s diseases (Fernández-Ruiz *et al.*, 2015 ▸). Furthermore, ABHD6 is differentially expressed in seven tumor cell lines, with particularly high expression being observed in Ewing family tumors (Lord *et al.*, 2013 ▸). Therefore, ABHD6 has been suggested as a new diagnostic marker for these tumors (Max *et al.*, 2009 ▸; Lord *et al.*, 2013 ▸). Moreover, ABHD6-knockout mice showed reduced body weight, improved glucose homeostasis and insulin action that prevents obesity and type 2 diabetes (Zhao *et al.*, 2016 ▸). ABHD6 has emerged as a promising pharmaceutical target, the inhibition of which has been studied biochemically and *in silico* (Feledziak *et al.*, 2012 ▸; Bowman & Makriyannis, 2013 ▸). However, the three-dimensional structure of ABHD6 remains elusive.

We recently characterized the α/β-hydrolase PA2949 from *P. aeruginosa* (Kovacic, Bleffert *et al.*, 2016 ▸). *P. aeruginosa* is a versatile pathogen that causes infections in mammals, plants and insects (Mahajan-Miklos *et al.*, 2000 ▸). It mainly infects immunocompromised patients, in particular cystic fibrosis, AIDS and cancer patients (Folkesson *et al.*, 2012 ▸). The pathogenicity of *P. aeruginosa* is predominantly related to the production of a large spectrum of cell-associated and cell-secreted virulence factors, among which are several α/β-hydrolases (Van Delden & Iglewski, 1998 ▸; Bofill *et al.*, 2010 ▸). PA2949 is a 34.8 kDa protein that shows esterase activity and is anchored to the *E. coli* membrane by a putative N-terminal transmembrane domain (Kovacic, Bleffert *et al.*, 2016 ▸). PA2949 contains a typical α/β-hydrolase Ser–His–Asp catalytic triad, as shown previously in our mutagenesis studies (Kovacic, Bleffert *et al.*, 2016 ▸).

Here, we report the expression in the homologous host *P. aeruginosa*, purification from membranes, crystallization and preliminary X-ray analysis of PA2949, a bacterial homolog of human ABHD6. To date, no structure is available of a protein containing an ABHD6-like domain. Therefore, the structure of the putatively single-pass integral membrane protein PA2949 should contribute to the understanding of the function of these proteins, with potential therapeutic implications.

## 2. Materials and methods   

### 2.1. Overexpression and purification   

*P. aeruginosa* PA01 cells harboring pBBR-*pa2949* (Kovacic, Bleffert *et al.*, 2016 ▸) were cultivated overnight in Luria–Bertani (LB) medium supplemented with tetracycline (100 µg ml^−1^) at 37°C (Table 1 ▸). This culture was used to inoculate an expression culture in LB medium supplemented with tetracycline (100 µg ml^−1^) to an initial OD_580 nm_ of 0.05. The cultures were grown at 37°C until they reached an OD_580 nm_ of ∼1. The cells were harvested by centrifugation (15 min at 6750*g* and 4°C), resuspended in 100 m*M* Tris–HCl buffer pH 8 and disrupted using a French press. PA2949 was purified from the membrane fraction by immobilized metal-affinity chromatography (IMAC) using Ni–NTA agarose (Qiagen, Hilden, Germany) as described previously (Kovacic, Bleffert *et al.*, 2016 ▸), with the modification that the Triton X-100 in the elution buffer was replaced by 30 m*M* *n*-octyl β-d-glucoside (OG). The PA2949 samples eluted from the Ni–NTA column were transferred into 100 m*M* Tris–HCl buffer pH 8 supplemented with 30 m*M* *n*-octyl β-d-glucoside (OG) by gel filtration using a PD-10 column (GE Healthcare, Solingen, Germany) according to the manufacturer’s protocol. This PA2949 sample was loaded onto an anion-exchange chromatography column containing UNOsphere Q medium (Bio-Rad Laboratories, Munich, Germany) equilibrated with 100 m*M* Tris–HCl pH 8 buffer containing 30 m*M* OG. Purified PA2949 was collected in the flowthrough fraction and the protein impurities were retained on the column. The purified PA2949 was stored at room temperature.

### 2.2. SDS–PAGE, preparation of antisera and immunodetection   

Proteins were analyzed by sodium dodecyl sulfate–polyacrylamide gel electrophoresis (SDS–PAGE) under denaturating conditions on 12%(*w*/*v*) gels as described by Laemmli (1970 ▸). SDS–PAGE slices containing PA2949 were used to raise polyclonal antisera in rabbits (Eurogentec, Seraing, Belgium) by injecting 100 µg antigen four times over three months. The proteins transferred from the SDS–PAGE gel to PVDF membranes by Western blotting (Yuen *et al.*, 1989 ▸) were detected using anti-PA2949 specific antiserum according to the following procedure. The PVDF membrane with transferred proteins was saturated for 1 h in 25 m*M* Tris–HCl buffer pH 8 containing 150 m*M* NaCl, 3 m*M* KCl, 0.2%(*v*/*v*) Tween-20 and 5%(*w*/*v*) skimmed milk, followed by incubation with anti-PA2949 antiserum diluted 5000 times with the same buffer as used for saturation. The membrane was washed three times with 25 m*M* Tris–HCl buffer pH 8 containing 150 m*M* NaCl, 3 m*M* KCl and 0.2%(*v*/*v*) Tween-20, incubated for 1 h with goat anti-rabbit immunoglobulin G antibodies coupled to HRP (horseradish peroxidase; Sigma, St Louis, USA) according to the manufacturer’s instructions, washed again three times with 25 m*M* Tris–HCl buffer pH 8 containing 150 m*M* NaCl, 3 m*M* KCl and 0.2%(*v*/*v*) Tween-20, and then exposed with an ECL Western blotting detection kit (Amersham Bioscience, Freiburg). The protein concentration was determined by measuring the *A*_280 nm_ using a NanoDrop 2000c spectrophotometer (ThermoFisher Scientific Inc., Waltham, Massachusetts, USA) and using an extinction coefficient (∊ = 22 920 *M* ^−1^ cm^−1^) for PA2949 with a His_6_ tag calculated using the *ProtParam* tool (Navia-Paldanius *et al.*, 2012 ▸).

### 2.3. Enzyme-activity assays   

Enzyme activities towards fatty-acid esters of *p*-nitrophenol (*p*-NP) were determined according to a previously described method (Kovacic, Bleffert *et al.*, 2016 ▸). The enzymatic reactions were performed in a 96-well microplate by adding 5 µl (8 n*M*) of enzyme sample to 150 µl of the substrate. The kinetic parameters *K*_m_ and *k*_cat_ were determined by measuring the PA2949 activity with 0.05. 0.1, 0.2, 0.3, 0.5 and 1 m*M* *p*-nitrophenyl butyrate; the data were fitted to the Michaelis–Menten equation using a nonlinear regression method.

Triacylglyceride (Sigma, Taufkirchen, Germany) and 2-AG (Avanti, Alabaster, USA) substrates were prepared for enzyme-activity assays (25 µl enzyme + 25 µl substrate) as described previously (Jaeger & Kovacic, 2014 ▸). The amount of fatty acids released by PA2949 was determined using a NEFA-HR(2) kit (Wako Chemicals, Neuss, Germany).

### 2.4. Thermal stability   

Fluorescence-based thermal stability experiments were performed using a Prometheus NT.48 from NanoTemper Technologies. Capillaries containing 10 µl PA2949 sample were inserted into the machine, the temperature was increased from 20 to 90°C at a rate of 1°C min^−1^, and the fluorescence was measured at emission wavelengths of 330 and 350 nm. Enzyme activity-based thermal unfolding experiments were performed by measuring the residual esterase activity of a PA2949 sample incubated for 1 h at temperatures from 30 to 70°C. The enzyme assay was performed as described above using *p*-NPC_4_ substrate. The ratio of fluorescence intensities at 350 and 330 nm (for the biophysical method) and esterase activities (for the biochemical method) as a function of temperature were used to determine the transition temperatures, which can be interpreted as the melting temperatures.

### 2.5. Crystallization and data collection   

For crystallization, purified PA2949 was concentrated to 3.5 mg ml^−1^ using an ultrafiltration device with a 30 kDa molecular-weight cutoff membrane. Initial crystallization screening was performed with the AmSO4, CubicPhase I and CubicPhase II, MbClass, PEGs I and PEGs II Suites (Qiagen, Hilden, Germany), Crystal Screen and Crystal Screen 2 (Hampton Research, Aliso Viejo, California, USA) and Wizard Screens 1 and 2 (Emerald BioStructures, Bainbridge Island, Washington, USA) crystallization screening kits at 19°C using the sitting-drop vapor-diffusion method (Table 2 ▸). The PA2949 crystals used for X-ray diffraction were grown from a solution consisting of 1 µl PA2949 solution and 1 µl reservoir solution [100 m*M* trisodium citrate pH 5.6 with 10%(*w*/*v*) PEG 4000 and 10%(*w*/*v*) propan-2-ol]. Crystals appeared within three weeks. Before cryocooling, single crystals were soaked stepwise in reservoir solution containing up to 15%(*w*/*v*) polyethylene glycol 200. The X-ray diffraction data were recorded on beamline ID23-1 at the European Synchrotron Radiation Facility, Grenoble, France using a wavelength of 0.9791 Å. The data-collection strategies, taking radiation damage into account, were based on calculations using *BEST* (Bourenkov & Popov, 2010 ▸). Data processing was carried out using *XDS* (Kabsch, 2010 ▸) and *AIMLESS* (which is part of the *CCP*4 software package; Winn *et al.*, 2011 ▸). Structure determination is currently being performed by molecular replacement.

## 3. Results and discussion   

### 3.1. Similarity of *P. aeruginosa* PA2949 and human ABHD6   

**Figure 1:**
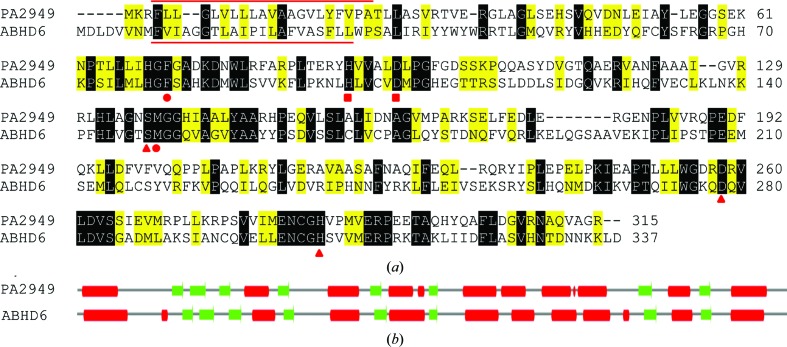
Sequence alignment of *P. aeruginosa* PA2949 with human ABHD6 and secondary-structure prediction. (*a*) Sequence alignment. The putative active site comprising the catalytic triad (Ser148, His306 and Asp278 in ABHD6, and Ser137, His286 and Asp258 in PA2949) and the oxyanion-hole residues (Phe80 and Met149 in ABHD6, and Phe71 and Met138 in PA2949) are indicated by red triangles and circles, respectively. Conserved residues (His99 and Asp104 in ABHD6 and His90 and Asp95 in PA2949) of the putative H*XXXX*D acyltransferase motif are indicated by red squares. The putative transmembrane domains of PA2949 (amino acids 4–24) and ABHD6 (amino acids 9–29) are indicated by red lines above and below the sequences, respectively. Identical and similar residues are shaded in black and yellow, respectively. The pairwise sequence alignment was generated with the *EMBOSS Water* (Rice *et al.*, 2000 ▸) local alignment tool from the European Bioinformatics Institute (EMBL–EBI). (*b*) Secondary structure was predicted with the *JPred*4 server (Drozdetskiy *et al.*, 2015 ▸) using a method based on hidden Markov models. α-Helices are indicated in red and β-sheets in green.

Previously, we have described *P. aeruginosa* PA2949 as a membrane-bound esterase that is homologous to bacterial esterases/lipases (Kovacic, Bleffert *et al.*, 2016 ▸). PA2949 shows 27% sequence identity and 49% sequence similarity to the human α/β-hydrolase domain 6 (ABHD6) protein, which exerts lipase activity (Blankman *et al.*, 2007 ▸; Fig. 1 ▸ *a*). Secondary-structure prediction revealed a similar organization of eight β-strands and ten α-helices in both proteins (Fig. 1 ▸ *b*). Sequence analysis revealed conservation of Ser137 in PA2949 and Ser148 in ABHD6, which yielded inactive enzymes when mutated to alanine (Kovacic, Bleffert *et al.*, 2016 ▸; Navia-Paldanius *et al.*, 2012 ▸). Inhibition experiments with PA2949 and ABHD6 revealed similar inhibition profiles with respect to the typical lipase inhibitor tetrahydrolipstatin (THL; Ašler *et al.*, 2007 ▸) and the arylesterase inhibitor phenylmethyl­sulfonyl fluoride (PMSF; Blankman *et al.*, 2007 ▸; Kovacic, Bleffert *et al.*, 2016 ▸). Furthermore, the other two putative members of the catalytic triad (His and Asp) and the putative oxyanion-hole residues (Met and Phe) are strictly conserved between the two enzymes (Fig. 1 ▸). The putative acyltransferase motif (H*XXXX*D, where *X* represents any residue) reported for ABHD6 (Lord *et al.*, 2013 ▸) is also conserved in PA2949 (Fig. 1 ▸ *a*). Although promiscuous enzymes with lipase and acyltransferase activities have previously been described (Brumlik & Buckley, 1996 ▸; Vijayaraj *et al.*, 2012 ▸), the acyltransferase activity of ABHD6 and PA2949 still needs to be demonstrated experimentally. Prediction of cellular localization revealed a putative N-terminal transmembrane (TM) domain (Fig. 1 ▸), which is likely to serve as a signal anchor, in both proteins (Kovacic, Bleffert *et al.*, 2016 ▸; Lord *et al.*, 2013 ▸). This suggestion is supported by the experimentally demonstrated membrane localization of PA2949 and ABHD6 (Blankman *et al.*, 2007 ▸; Kovacic, Bleffert *et al.*, 2016 ▸). To conclude, comparison of the sequence properties of PA2949 and ABHD6 revealed substantial similarity despite the considerable evolutionary distance between them.

### 3.2. Optimized expression and purification procedure for *P. aeruginosa* PA2949   

**Figure 2:**
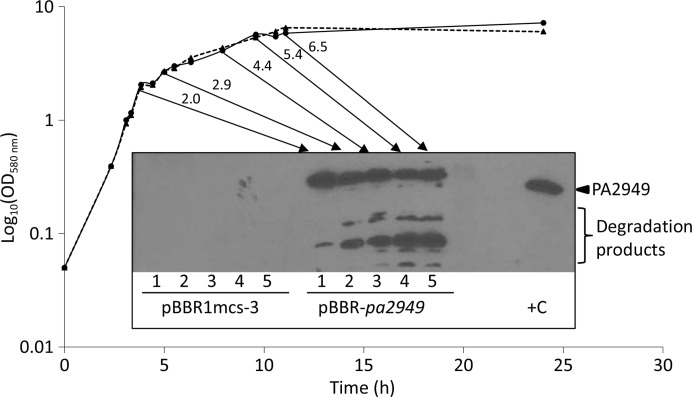
Expression of PA2949 in *P. aeruginosa*. *P. aeruginosa* PA01 strain carrying pBBR1mcs-3 (empty-vector control, dashed line) or pBBR-*pa2949* (solid line) was grown in LB medium at 37°C; cells were collected at five (1–5) different growth stages (the respective OD_580 nm_ values are indicated above the arrows). Cells equivalent to 100 µl of culture with an OD_580 nm_ of 1 were analyzed by SDS–PAGE and the presence of PA2949 in each sample was tested by Western blotting using anti-His-tag antibodies. Purified PA2949 (+C) was used as a positive control.

For the crystallographic characterization of PA2949, we attempted to purify milligram amounts of PA2949 under conditions where it retains activity during the time frame of the crystallization experiments. Our previously published system for the expression of PA2949 in *E. coli* BL21(DE3) cells (Kovacic, Bleffert *et al.*, 2016 ▸) and purification by immobilized metal-affinity chromatography in the presence of Triton X-100 yielded a protein that showed a loss of enzymatic activity after storage at 4 or −20°C in the presence or absence of glycerol (data not shown). To overcome the protein stability issue, we tested whether the homologous host *P. aeruginosa* might be more suitable for the expression of membrane-bound PA2949 than a heterologous host. We developed the *P. aeruginosa* PA01 system for induction-independent constitutive expression of PA2949 controlled by a moderate *lac* promoter on the pBBR1mcs-3 plasmid. *P. aeruginosa* PA01 cells carrying the pBBR-*pa2949* plasmid (Kovacic, Bleffert *et al.*, 2016 ▸) expressed catalytically active PA2949 in the late logarithmic and stationary phases. While the esterase activity was constant during growth, degradation of intracellular PA2949 in the stationary-phase culture was observed as judged from Western blot analysis (Fig. 2 ▸). Consequently, for purification purposes cells were harvested in the late log­arithmic phase (an OD_580 nm_ of ∼1) to avoid the degradation of PA2949 observed in cultures with an OD_580 nm_ of 2 (Fig. 2 ▸).

**Figure 3:**
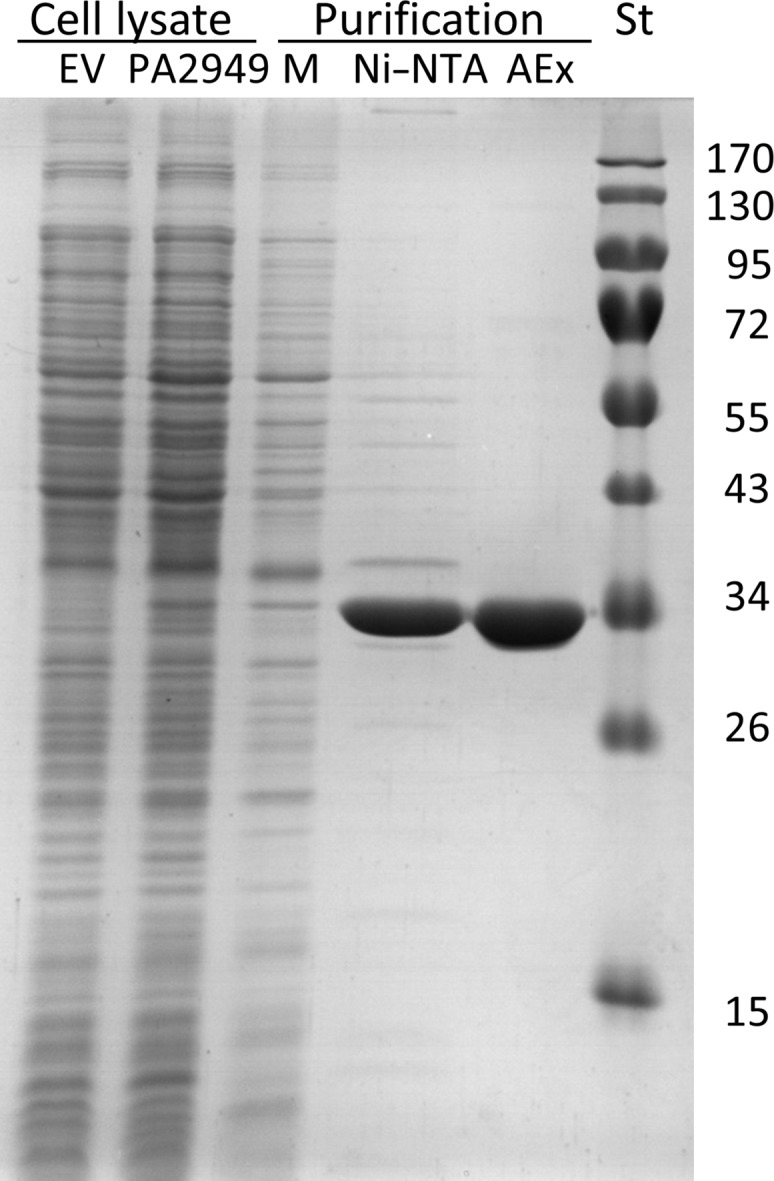
Purification of PA2949 from *P. aeruginosa*. (*a*) *P. aeruginosa* PA01 cells carrying the *pa2949* expression vector (pBBR-*pa2949*) or pBBR1mcs-3 (EV, empty vector) were harvested at an OD_580 nm_ of 1 and disrupted. Membranes (M) were separated by ultracentrifugation. PA2949 was purified with an Ni–NTA column and subsequently with a UNOsphere Q anion-exchange column (AEx) in the presence of *n*-octyl β-d-glucoside. The samples were analyzed by SDS–PAGE stained with Coomassie Brilliant Blue G-250. The molecular weights of protein standards (St) are indicated on the right in kDa.

**Figure 4:**
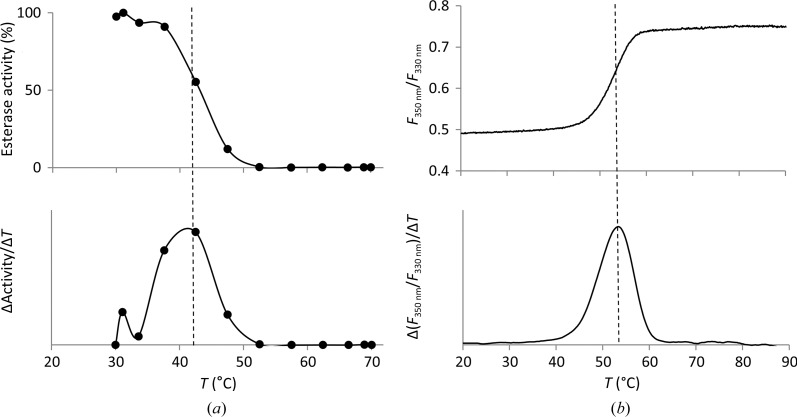
Thermal stability of PA2949 in OG buffer measured by determination of enzymatic activity (*a*) and change in intrinsic fluorescence (*b*). The relative esterase activity and the derivative of esterase activity (ΔActivity/Δ*T*) as a function of temperature are shown in the upper and lower panels, respectively. 100% activity corresponds to the highest measured activity of PA2949. The fluorescence ratio (*F*_350 nm_/*F*_330 nm_) and the derivative of the fluorescence ratio [Δ(*F*_350 nm_/*F*_330 nm_)/Δ*T*] as a function of temperature are shown in the upper and lower diagrams, respectively. Dashed lines indicate calculated melting temperatures. Enzyme-activity results represent measurements of three independent experiments each with at least three samples. Standard deviations were within 10%. The fluorescence-based unfolding result is a representative single curve.

The selection of a detergent to provide stable conditions for membrane proteins after their extraction from bacterial membranes is essential for subsequent characterization. The mild, non-ionic detergent Triton X-100, widely used for solubilizing proteins from membranes (Schnaitman, 1971 ▸; Slinde & Flatmark, 1976 ▸), showed a good performance in solubilizing *P. aeruginosa* membranes (Kovacic, Bleffert *et al.*, 2016 ▸). However, denaturation of a membrane protein can be caused by Triton X-100 owing to suboptimal stabilization of the TM domains (Seddon *et al.*, 2004 ▸; Garavito & Ferguson-Miller, 2001 ▸), destabilization of extramembranous soluble domains (Yang *et al.*, 2014 ▸) or by reactive peroxides that are products of the oxidation and hydrolysis of Triton X-100 (Ashani & Catravas, 1980 ▸; Moraes *et al.*, 2014 ▸). For these reasons, we applied the mild, non-ionic detergent OG, commonly used in membrane-protein research (Moraes *et al.*, 2014 ▸; Arolas *et al.*, 2014 ▸), to PA2949. The PA2949 sample eluted from the IMAC column using a buffer that contains OG showed the presence of other protein impurities (Fig. 3 ▸), as also observed for purification from *E. coli* (Kovacic, Bleffert *et al.*, 2016 ▸). We next purified PA2949 by anion-exchange chromatography to a homogeneity that was sufficient for further biochemical and crystallographic studies (Fig. 3 ▸). The OG-stabilized PA2949 protein retained more than 56% of its esterase activity, as measured with *p*-nitrophenyl (*p*-NP) hexanoate (C_6_) substrate, after storage at room temperature for six months. The thermal stability of PA2949 in OG was studied by measuring the change in intrinsic protein fluorescence upon thermal unfolding and by measuring the residual esterase activity after the exposure of PA2949 to different temperatures. These experiments revealed melting temperatures of PA2949 of 43.4 ± 0.5 and 53.1 ± 0.4°C calculated from enzyme-activity and fluorescence measurements, respectively (Fig. 4 ▸). It is likely that differences in the experimental setup (1 h of incubation in the enzymatic method *versus* continuous heating in the nanoDSF method) led to the observed discrepancy in the melting temperatures.

### 3.3. Substrate specificity of *P. aeruginosa* PA2949   

**Figure 5:**
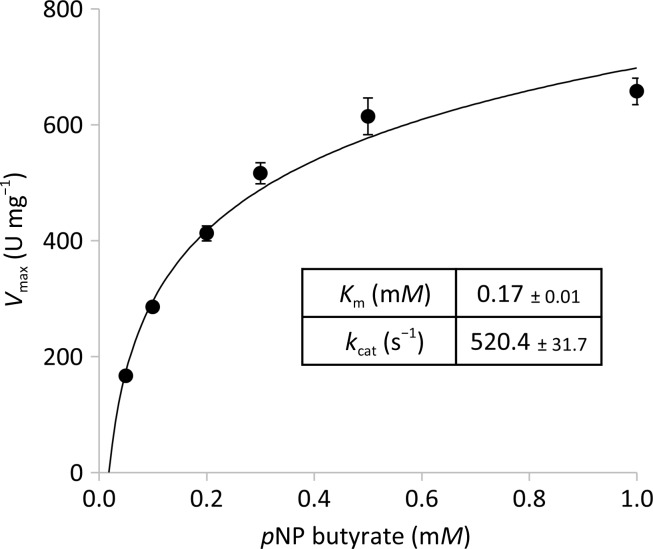
Enzyme kinetics of PA2949. The esterase activity of PA2949 (8 n*M*) purified with OG was measured using *p*NP C_4_, and kinetic parameters (± standard deviation) were determined by nonlinear regression analysis of data fitted to the Michaelis–Menten equation. The results represent measurements from eight independent experiments.

PA2949 purified from *P. aeruginosa* in the presence of OG showed a 2.3-fold higher esterase activity than the enzyme purified from *E. coli* with Triton X-100 (198.8 ± 5.1 U mg^−1^; Kovacic, Bleffert *et al.*, 2016 ▸) using *p*-NPC_6_ as a substrate (Table 3 ▸). Substrate-specificity measurements revealed the highest activity of PA2949 to be with *p*-NP octanoate (C_8_), and that the activity declines with increasing length of the fatty-acid acyl chain to reach no measurable activity with *p*-NP stearate (C_18_) (Table 3 ▸). PA2949 hydrolyses natural triacyl­glycerol substrates with a similar specificity as *p*-NP esters, although its activity with triglycerides was lower (Table 3 ▸). Apparently, PA2949 shows a typical activity profile for esterases that are capable of releasing medium-chain fatty acids from water-soluble carboxylic esters (Leščic Ašler *et al.*, 2010 ▸; Kovacic, Mandrysch *et al.*, 2016 ▸; Chow *et al.*, 2012 ▸; Ali *et al.*, 2012 ▸). Kinetics measurements of the hydrolysis of a typical esterase substrate, *p*-NP butyrate (C_4_), revealed that PA2949 follows Michaelis–Menten kinetics, as reported for most esterases (Fig. 5 ▸). Interestingly, ABHD6 also hydrolyses TAG substrates and shows a preference for esters with medium-chain acyl chains (from C_8_ to C_14_; Navia-Paldanius *et al.*, 2012 ▸). However, *in vitro* measurements showed a high activity of ABHD6 against the natural substrate 2-AG, which contains a long-chain fatty acid of 20 C atoms and four unsaturated bonds (Navia-Paldanius *et al.*, 2012 ▸). Our results with purified PA2949 demonstrated that the enzyme rapidly releases the fatty acid from the 2-AG substrate with an activity of 12.2 ± 0.3 nanomoles of fatty acid per milligram of PA2949 per minute. This activity is in the region of the activity reported for ABHD6 (7.4 ± 0.5 nanomoles of fatty acid per milligram of ABHD6 per minute), although these measurements were performed with lysates of human cells transiently expressing ABHD6 (Navia-Paldanius *et al.*, 2012 ▸). To conclude, the substrate profiling of PA2949 suggests that PA2949 and ABHD6 are biochemically similar, which is in agreement with the sequence similarity and the similarities in their inhibition results and cellular localization. Although the role of arachidonic acid as a precursor of signaling messengers in eukary­otes has been established, limited data are available on its presence and function in prokaryotes (Bajpai & Bajpai, 1992 ▸; Martínez & Campos-Gómez, 2016 ▸). The physiological relevance of the 2-AG hydrolase activity of PA2949 still needs to be elucidated.

### 3.4. Crystallization of PA2949   

**Figure 6:**
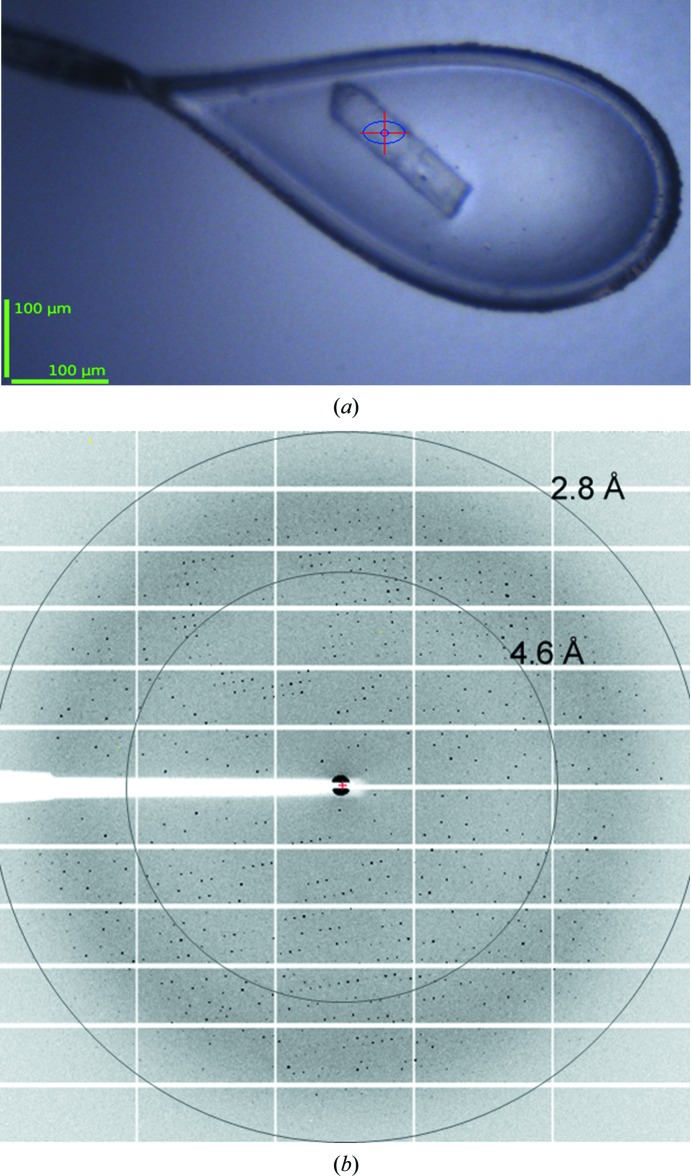
Crystal and initial X-ray diffraction pattern of PA2949. (*a*) A tetragonal PA2949 crystal in a loop prepared for collection of the diffraction pattern. (*b*) X-ray diffraction pattern from a reference set of two images taken with a resolution of ∼2.8 Å (edge of the detector) with a maximum φ separation of 90° with Δφ = 1°. The optimal strategy for data collection was determined based on these images using *MOSFLM* (Winn *et al.*, 2011 ▸) and *BEST* (Bourenkov & Popov, 2010 ▸). The given resolution for data collection was 2.5 Å (for results, see Table 4 ▸).

Proteins with a single TM domain are considered to be difficult to crystallize, which is the reason why few structures of them are available (Monk *et al.*, 2014 ▸; Ray *et al.*, 2018 ▸). The concentrated PA2949 sample (3.5 mg ml^−1^) was centrifuged (21 000*g*, 10 min) prior to performing crystallization setups. Single crystals were obtained using the sitting-drop vapor-diffusion method at 19°C after a few rounds of fine screening around the buffer conditions described in Section 2. Tetragonal crystals of approximate dimensions 180 × 40 × 40 µm grew after an equilibration period of three weeks (Fig. 6 ▸). These crystals diffracted to 2.54 Å resolution. Data processing revealed the space group to be *I*4_1_22, with unit-cell parameters *a* = *b* = 135.36, *c* = 205.29 Å, α = β = γ = 90°. A summary of the X-ray crystallographic data-collection statistics is presented in Table 4 ▸. The calculated Matthews coefficient and the solvent content were 2.9 Å^3^ Da^−1^ and 58%, respectively. This suggests the presence of two molecules in the asymmetric unit of the PA2949 crystals. Currently, we are fine-tuning the crystallization conditions to improve the quality of the crystals for successful structure determination using the molecular-replacement method. The high-resolution crystal structure of PA2949 will provide information on the α/β-hydrolase class of proteins, which could be important in order to understand their structure–function relationship.

## Figures and Tables

**Table 1:** PA2949 production information

Source organism	*P. aeruginosa* PA01
DNA source	*P. aeruginosa* PA01
Forward primer (5′–3′)†	AAACATATGAAACGATTCCTC
Reverse primer (5′–3′)†	TCAGAGCTC **CACCACCACCACCACCAC**GCGACCGGCCAC
Cloning vector	pBBR1mcs-3
Expression vector	pBBR-*pa2949* (Kovacic, Bleffert *et al.*, 2016 ▸)
Cloning host	*E. coli* DH5α
Expression host	*P. aeruginosa* PA01
PA2949-His_6_ amino-acid sequence	MKRFLLGLVLLLAVAAGVLYFVPATLLASVRTVERGLAGLSEHSVQVDNLEIAYLEGGSEKNPTLLLIHGFGADKDNWLRFARPLTERYHVVALDLPGFGDSSKPQQASYDVGTQAERVANFAAAIGVRRLHLAGNSMGGHIAALYAARHPEQVLSLALIDNAGVMPARKSELFEDLERGENPLVVRQPEDFQKLLDFVFVQQPPLPAPLKRYLGERAVAASAFNAQIFEQLRQRYIPLEPELPKIEAPTLLLWGDRDRVLDVSSIEVMRPLLKRPSVVIMENCGHVPMVERPEETAQHYQAFLDGVRNAQVAGRHHHHHHEL

† The sequences encoding NdeI and SacI restriction sites are underlined and that encoding the C-terminal His_6_ tag is in bold.

**Table 2:** Crystallization conditions

Method	Sitting-drop vapor diffusion
Plate type	NeXtal Evolution μplate (Qiagen, Hilden, Germany)
Temperature (°C)	19
Protein concentration (mg ml^−1^)	3.5
Buffer composition of protein solution	100 m*M* Tris–HCl pH 8, 30 m*M* OG
Composition of reservoir solution	100 m*M* trisodium citrate pH 5.6 with 10%(*w*/*v*) PEG 4000 and 10%(*w*/*v*) propan-2-ol
Volume, ratio of drop	2 µl, 1:1
Volume of reservoir (µl)	70

**Table 3:** Substrate specificities of PA2949 with *p*-nitrophenyl (*p*-NP) esters and triacylglycerides (TAG)

Acyl-chain length	*p*-NP activity† (U mg^−1^)	TAG activity† (mU mg^−1^)
C_2_	108.9 ± 6.5	NA
C_4_	520.4 ± 31.7	NA
C_6_	458.0 ± 11.5	10.1 ± 0.2
C_8_	621.2 ± 34.2	ND
C_10_	351.3 ± 25.5	12.6 ± 0.2
C_12_	162.5 ± 12.5	3.9 ± 0.2
C_14_	58.9 ± 19.4	0.3 ± 0.1
C_16_	18.4 ± 5.1	NA
C_18_	NA	NA

† All values are the mean ± standard deviation of three independent measurements of each set in triplicate. NA, not active; ND, not determined.

**Table 4:** X-ray diffraction data-collection and processing statistics for PA2949 Values in parentheses are for the highest resolution shell.

Beamline	ID23-1
Detector	PILATUS 6M
Wavelength (Å)	0.9791
Resolution range (Å)	47.86–2.54 (2.65–2.54)
Space group	*I*4_1_22
*a*, *b*, *c* (Å)	135.36, 135.36, 205.29
α, β, γ (°)	90, 90, 90
Total reflections	179853
Unique reflections	31637 (3807)
Multiplicity	5.7 (5.9)
Completeness (%)	99.7 (99.9)
Mean *I*/σ(*I*)	8.8 (1.3)
Wilson *B* factor (Å^2^)	45.91
*R*_merge_	0.148 (1.656)
*R*_meas_	0.162 (1.813)
*R*_p.i.m._	0.067 (0.730)
Mn(*I*) half-set correlation CC_1/2_	0.994 (0.419)
Expected No. of molecules in asymmetric unit	2
Solvent fraction (%)	58
Matthews coefficient (Å^3^ Da^−1^)	2.9

## References

[bb1] Ali, Y. B., Verger, R. & Abousalham, A. (2012). *Methods Mol. Biol.* **861**, 31–51. 10.1007/978-1-61779-600-5_2 22426710

[bb2] Arolas, J. L., García-Castellanos, R., Goulas, T., Akiyama, Y. & Gomis-Rüth, F. X. (2014). *Protein Expr. Purif.* **99**, 113–118. 10.1016/j.pep.2014.04.008 24769134

[bb3] Ashani, Y. & Catravas, G. N. (1980). *Anal. Biochem.* **109**, 55–62. 10.1016/0003-2697(80)90009-3 7469018

[bb4] Ašler, I. L., Zehl, M., Kovačić, F., Müller, R., Abramić, M., Allmaier, G. & Kojić-Prodić, B. (2007). *Biochim. Biophys. Acta*, **1770**, 163–170. 10.1016/j.bbagen.2006.10.011 17137716

[bb5] Bajpai, P. & Bajpai, P. K. (1992). *Biotechnol. Appl. Biochem.* **15**, 1–10. 10.1111/j.1470-8744.1992.tb00194.x 1550658

[bb6] Blankman, J. L., Simon, G. M. & Cravatt, B. F. (2007). *Chem. Biol.* **14**, 1347–1356. 10.1016/j.chembiol.2007.11.006 PMC2692834 18096503

[bb7] Bofill, C., Prim, N., Mormeneo, M., Manresa, A., Javier Pastor, F. I. & Diaz, P. (2010). *Biochimie*, **92**, 307–316. 10.1016/j.biochi.2009.11.005 19944735

[bb8] Bourenkov, G. P. & Popov, A. N. (2010). *Acta Cryst.* D**66**, 409–419. 10.1107/S0907444909054961 PMC2852305 20382994

[bb9] Bowman, A. L. & Makriyannis, A. (2013). *Chem. Biol. Drug Des.* **81**, 382–388. 10.1111/cbdd.12086 PMC3573238 23110439

[bb10] Brumlik, M. J. & Buckley, J. T. (1996). *J. Bacteriol.* **178**, 2060–2064. 10.1128/jb.178.7.2060-2064.1996 PMC177905 8606184

[bb11] Chow, J., Kovacic, F., Dall Antonia, Y., Krauss, U., Fersini, F., Schmeisser, C., Lauinger, B., Bongen, P., Pietruszka, J., Schmidt, M., Menyes, I., Bornscheuer, U. T., Eckstein, M., Thum, O., Liese, A., Mueller-Dieckmann, J., Jaeger, K. E. & Streit, W. R. (2012). *PLoS One*, **7**, e47665. 10.1371/journal.pone.0047665 PMC3480424 23112831

[bb12] Drozdetskiy, A., Cole, C., Procter, J. & Barton, G. J. (2015). *Nucleic Acids Res.* **43**, W389–W394. 10.1093/nar/gkv332 PMC4489285 25883141

[bb13] Feledziak, M., Lambert, D. M., Marchand-Brynaert, J. & Muccioli, G. G. (2012). *Recent Pat. CNS Drug. Discov.* **7**, 49–70. 10.2174/157488912798842223 22280341

[bb14] Fernández-Ruiz, J., Romero, J. & Ramos, J. A. (2015). *Handb. Exp. Pharmacol.* **231**, 233–259. 10.1007/978-3-319-20825-1_8 26408163

[bb15] Flores-Díaz, M., Monturiol-Gross, L., Naylor, C., Alape-Girón, A. & Flieger, A. (2016). *Microbiol. Mol. Biol. Rev.* **80**, 597–628. 10.1128/MMBR.00082-15 PMC4981679 27307578

[bb16] Folkesson, A., Jelsbak, L., Yang, L., Johansen, H. K., Ciofu, O., Høiby, N. & Molin, S. (2012). *Nat. Rev. Microbiol.* **10**, 841–851. 10.1038/nrmicro2907 23147702

[bb17] Garavito, R. M. & Ferguson-Miller, S. (2001). *J. Biol. Chem.* **276**, 32403–32406. 10.1074/jbc.R100031200 11432878

[bb18] Heikinheimo, P., Goldman, A., Jeffries, C. & Ollis, D. L. (1999). *Structure*, **7**, R141–R146. 10.1016/s0969-2126(99)80079-3 10404588

[bb19] Jaeger, K. E. & Kovacic, F. (2014). *Methods Mol. Biol.* **1149**, 111–134. 10.1007/978-1-4939-0473-0_12 24818902

[bb20] Kabsch, W. (2010). *Acta Cryst.* D**66**, 125–132. 10.1107/S0907444909047337 PMC2815665 20124692

[bb21] Kovacic, F., Bleffert, F., Caliskan, M., Wilhelm, S., Granzin, J., Batra-Safferling, R. & Jaeger, K. E. (2016). *FEBS Open Bio*, **6**, 484–493. 10.1002/2211-5463.12061 PMC4856427 27419054

[bb22] Kovacic, F., Mandrysch, A., Poojari, C., Strodel, B. & Jaeger, K. E. (2016). *Protein Eng. Des. Sel.* **29**, 65–76. 10.1093/protein/gzv061 PMC5943684 26647400

[bb23] Laemmli, U. K. (1970). *Nature (London)*, **227**, 680–685. 10.1038/227680a0 5432063

[bb24] Leščić Ašler, I., Ivić, N., Kovačić, F., Schell, S., Knorr, J., Krauss, U., Wilhelm, S., Kojić-Prodić, B. & Jaeger, K.-E. (2010). *ChemBioChem*, **11**, 2158–2167. 10.1002/cbic.201000398 20931591

[bb25] Lord, C. C., Thomas, G. & Brown, J. M. (2013). *Biochim. Biophys. Acta*, **1831**, 792–802. 10.1016/j.bbalip.2013.01.002 PMC4765316 23328280

[bb26] Mahajan-Miklos, S., Rahme, L. G. & Ausubel, F. M. (2000). *Mol. Microbiol.* **37**, 981–988. 10.1046/j.1365-2958.2000.02056.x 10972817

[bb27] Marrs, W. R., Blankman, J. L., Horne, E. A., Thomazeau, A., Lin, Y. H., Coy, J., Bodor, A. L., Muccioli, G. G., Hu, S. S.-J., Woodruff, G., Fung, S., Lafourcade, M., Alexander, J. P., Long, J. Z., Li, W., Xu, C., Möller, T., Mackie, K., Manzoni, O. J., Cravatt, B. F. & Stella, N. (2010). *Nat. Neurosci.* **13**, 951–957. 10.1038/nn.2601 PMC2970523 20657592

[bb28] Martínez, E. & Campos-Gómez, J. (2016). *Nat. Commun.* **7**, 13823. 10.1038/ncomms13823 PMC5155153 27929111

[bb29] Max, D., Hesse, M., Volkmer, I. & Staege, M. S. (2009). *Cancer Sci.* **100**, 2383–2389. 10.1111/j.1349-7006.2009.01347.x PMC11158961 19793082

[bb30] Monk, B. C., Tomasiak, T. M., Keniya, M. V., Huschmann, F. U., Tyndall, J. D., O’Connell, J. D., Cannon, R. D., McDonald, J. G., Rodriguez, A., Finer-Moore, J. S. & Stroud, R. M. (2014). *Proc. Natl Acad. Sci. USA*, **111**, 3865–3870. 10.1073/pnas.1324245111 PMC3956205 24613931

[bb31] Moraes, I., Evans, G., Sanchez-Weatherby, J., Newstead, S. & Shaw Stewart, P. D. (2014). *Biochim. Biophys. Acta*, **1838**, 78–87. 10.1016/j.bbamem.2013.07.010 PMC3898769 23860256

[bb32] Navia-Paldanius, D., Savinainen, J. R. & Laitinen, J. T. (2012). *J. Lipid Res.* **53**, 2413–2424. 10.1194/jlr.M030411 PMC3466009 22969151

[bb33] Pérez, D., Kovacic, F., Wilhelm, S., Jaeger, K. E., García, M. T., Ventosa, A. & Mellado, E. (2012). *Microbiology*, **158**, 2192–2203. 10.1099/mic.0.058792-0 22609754

[bb34] Rauwerdink, A. & Kazlauskas, R. J. (2015). *ACS Catal.* **5**, 6153–6176. 10.1021/acscatal.5b01539 PMC5455348 28580193

[bb35] Ray, L. C., Das, D., Entova, S., Lukose, V., Lynch, A. J., Imperiali, B. & Allen, K. N. (2018). *Nat. Chem. Biol.* **14**, 538–541. 10.1038/s41589-018-0054-z PMC6202225 29769739

[bb36] Rice, P., Longden, I. & Bleasby, A. (2000). *Trends Genet.* **16**, 276–277. 10.1016/s0168-9525(00)02024-2 10827456

[bb37] Schnaitman, C. A. (1971). *J. Bacteriol.* **108**, 545–552. 10.1128/jb.108.1.545-552.1971 PMC247096 4941569

[bb38] Seddon, A. M., Curnow, P. & Booth, P. J. (2004). *Biochim. Biophys. Acta*, **1666**, 105–117. 10.1016/j.bbamem.2004.04.011 15519311

[bb39] Slinde, E. & Flatmark, T. (1976). *Biochim. Biophys. Acta*, **455**, 796–805. 10.1016/0005-2736(76)90049-3 187245

[bb40] Van Delden, C. & Iglewski, B. H. (1998). *Emerg. Infect. Dis.* **4**, 551–560. 10.3201/eid0404.980405 PMC2640238 9866731

[bb41] Vijayaraj, P., Jashal, C. B., Vijayakumar, A., Rani, S. H., Venkata Rao, D. K. & Rajasekharan, R. (2012). *Plant Physiol.* **160**, 667–683. 10.1104/pp.112.202135 PMC3461547 22915575

[bb42] Winn, M. D., Ballard, C. C., Cowtan, K. D., Dodson, E. J., Emsley, P., Evans, P. R., Keegan, R. M., Krissinel, E. B., Leslie, A. G. W., McCoy, A., McNicholas, S. J., Murshudov, G. N., Pannu, N. S., Potterton, E. A., Powell, H. R., Read, R. J., Vagin, A. & Wilson, K. S. (2011). *Acta Cryst.* D**67**, 235–242. 10.1107/S0907444910045749 PMC3069738 21460441

[bb43] Yang, Z., Wang, C., Zhou, Q., An, J., Hildebrandt, E., Aleksandrov, L. A., Kappes, J. C., DeLucas, L. J., Riordan, J. R., Urbatsch, I. L., Hunt, J. F. & Brouillette, C. G. (2014). *Protein Sci.* **23**, 769–789. 10.1002/pro.2460 PMC4093953 24652590

[bb44] Yuen, S. W., Chui, A. H., Wilson, K. J. & Yuan, P. M. (1989). *Biotechniques*, **7**, 74–83. 2629835

[bb45] Zhao, S., Mugabo, Y., Ballentine, G., Attane, C., Iglesias, J., Poursharifi, P., Zhang, D., Nguyen, T. A., Erb, H., Prentki, R., Peyot, M.-L., Joly, E., Tobin, S., Fulton, S., Brown, J. M., Madiraju, S. R. M. & Prentki, M. (2016). *Cell Rep.* **14**, 2872–2888. 10.1016/j.celrep.2016.02.076 26997277

